# Supplementary material for: Pyruvate Dehydrogenase Kinase 1 inhibition mediated oxidative phosphorylation enhancement in cartilage promotes osteoarthritis progression
Source: BMC Musculoskelet Disord. 2023 Jul 20;24:597. doi: 10.1186/s12891-023-06585-6 (PMC10357736; doi:10.1186/s12891-023-06585-6)
Supplement: Supplementary file 1 — Additional file 1. [file 12891_2023_6585_MOESM1_ESM.zip › BP.Gsea.1653623667859/GOBP_TRICARBOXYLIC_ACID_CYCLE.html]

Details for gene set GOBP\_TRICARBOXYLIC\_ACID\_CYCLE[GSEA]

|  || Dataset | 55457\_collapsed\_to\_symbols.cls.cls #OA\_versus\_normal.cls.cls #OA\_versus\_normal\_repos |
| Phenotype | cls.cls#OA\_versus\_normal\_repos |
| Upregulated in class | OA |
| GeneSet | GOBP\_TRICARBOXYLIC\_ACID\_CYCLE |
| Enrichment Score (ES) | 0.43266043 |
| Normalized Enrichment Score (NES) | 1.5421147 |
| Nominal p-value | 0.014669927 |
| FDR q-value | 0.3133738 |
| FWER p-Value | 1.0 |
Table: GSEA Results Summary

  

Fig 1: Enrichment plot: GOBP\_TRICARBOXYLIC\_ACID\_CYCLE      
 Profile of the Running ES Score & Positions of GeneSet Members on the Rank Ordered List

  

| SYMBOL | TITLE | RANK IN GENE LIST | RANK METRIC SCORE | RUNNING ES | CORE ENRICHMENT || 1 | PDHB | pyruvate dehydrogenase E1 subunit beta [Source:HGNC Symbol;Acc:HGNC:8808] | 149 | 0.862 | 0.0766 | Yes |
| 2 | MDH1 | malate dehydrogenase 1 [Source:HGNC Symbol;Acc:HGNC:6970] | 271 | 0.772 | 0.1461 | Yes |
| 3 | ACO2 | aconitase 2 [Source:HGNC Symbol;Acc:HGNC:118] | 290 | 0.756 | 0.2221 | Yes |
| 4 | MDH2 | malate dehydrogenase 2 [Source:HGNC Symbol;Acc:HGNC:6971] | 615 | 0.618 | 0.2601 | Yes |
| 5 | IDH2 | isocitrate dehydrogenase (NADP(+)) 2 [Source:HGNC Symbol;Acc:HGNC:5383] | 967 | 0.525 | 0.2865 | Yes |
| 6 | SUCLG2 | succinate-CoA ligase GDP-forming subunit beta [Source:HGNC Symbol;Acc:HGNC:11450] | 1472 | 0.432 | 0.2916 | Yes |
| 7 | SDHB | succinate dehydrogenase complex iron sulfur subunit B [Source:HGNC Symbol;Acc:HGNC:10681] | 1818 | 0.382 | 0.3038 | Yes |
| 8 | IREB2 | iron responsive element binding protein 2 [Source:HGNC Symbol;Acc:HGNC:6115] | 2215 | 0.337 | 0.3075 | Yes |
| 9 | SDHA | succinate dehydrogenase complex flavoprotein subunit A [Source:HGNC Symbol;Acc:HGNC:10680] | 2231 | 0.335 | 0.3406 | Yes |
| 10 | SUCLG1 | succinate-CoA ligase GDP/ADP-forming subunit alpha [Source:HGNC Symbol;Acc:HGNC:11449] | 2269 | 0.330 | 0.3715 | Yes |
| 11 | NNT | nicotinamide nucleotide transhydrogenase [Source:HGNC Symbol;Acc:HGNC:7863] | 2425 | 0.311 | 0.3913 | Yes |
| 12 | PDHA1 | pyruvate dehydrogenase E1 subunit alpha 1 [Source:HGNC Symbol;Acc:HGNC:8806] | 2433 | 0.310 | 0.4225 | Yes |
| 13 | DHTKD1 | dehydrogenase E1 and transketolase domain containing 1 [Source:HGNC Symbol;Acc:HGNC:23537] | 3112 | 0.239 | 0.3942 | Yes |
| 14 | SDHC | succinate dehydrogenase complex subunit C [Source:HGNC Symbol;Acc:HGNC:10682] | 3145 | 0.235 | 0.4157 | Yes |
| 15 | SUCLA2 | succinate-CoA ligase ADP-forming subunit beta [Source:HGNC Symbol;Acc:HGNC:11448] | 3228 | 0.228 | 0.4327 | Yes |
| 16 | SDHD | succinate dehydrogenase complex subunit D [Source:HGNC Symbol;Acc:HGNC:10683] | 4055 | 0.151 | 0.3839 | No |
| 17 | IDH3G | isocitrate dehydrogenase (NAD(+)) 3 non-catalytic subunit gamma [Source:HGNC Symbol;Acc:HGNC:5386] | 4504 | 0.113 | 0.3606 | No |
| 18 | DLAT | dihydrolipoamide S-acetyltransferase [Source:HGNC Symbol;Acc:HGNC:2896] | 5647 | 0.020 | 0.2740 | No |
| 19 | FH | fumarate hydratase [Source:HGNC Symbol;Acc:HGNC:3700] | 5729 | 0.013 | 0.2690 | No |
| 20 | IDH1 | isocitrate dehydrogenase (NADP(+)) 1 [Source:HGNC Symbol;Acc:HGNC:5382] | 7109 | -0.089 | 0.1710 | No |
| 21 | OGDH | oxoglutarate dehydrogenase [Source:HGNC Symbol;Acc:HGNC:8124] | 8503 | -0.194 | 0.0826 | No |
| 22 | PDHA2 | pyruvate dehydrogenase E1 subunit alpha 2 [Source:HGNC Symbol;Acc:HGNC:8807] | 9124 | -0.241 | 0.0591 | No |
| 23 | ACO1 | aconitase 1 [Source:HGNC Symbol;Acc:HGNC:117] | 9426 | -0.266 | 0.0630 | No |
| 24 | IDH3A | isocitrate dehydrogenase (NAD(+)) 3 catalytic subunit alpha [Source:HGNC Symbol;Acc:HGNC:5384] | 10426 | -0.364 | 0.0226 | No |
| 25 | DLST | dihydrolipoamide S-succinyltransferase [Source:HGNC Symbol;Acc:HGNC:2911] | 10634 | -0.386 | 0.0460 | No |
| 26 | CS | citrate synthase [Source:HGNC Symbol;Acc:HGNC:2422] | 10748 | -0.399 | 0.0780 | No |
| 27 | OGDHL | oxoglutarate dehydrogenase L [Source:HGNC Symbol;Acc:HGNC:25590] | 10754 | -0.399 | 0.1184 | No |
| 28 | IDH3B | isocitrate dehydrogenase (NAD(+)) 3 non-catalytic subunit beta [Source:HGNC Symbol;Acc:HGNC:5385] | 11304 | -0.472 | 0.1240 | No |
Table: GSEA details [plain text format]

  

Fig 2: GOBP\_TRICARBOXYLIC\_ACID\_CYCLE      
 Blue-Pink O' Gram in the Space of the Analyzed GeneSet

  

Fig 3: GOBP\_TRICARBOXYLIC\_ACID\_CYCLE: Random ES distribution      
 Gene set null distribution of ES for **GOBP\_TRICARBOXYLIC\_ACID\_CYCLE**
